# Burden of chronic obstructive pulmonary disease in Ghana and globally from 1990 to 2021, with projections through 2050: a systematic analysis based on the Global Burden of Disease Study 2021

**DOI:** 10.3389/fmed.2025.1681411

**Published:** 2025-11-03

**Authors:** Emmanuel Mensah, Min Liu, Lingling Pan, Wei Lu, Susheng Zhou, Liqin Zhang, Yusheng Cheng, Shuoshuo Wei, Lei Zha

**Affiliations:** ^1^Department of Pulmonary and Critical Care Medicine, The First Affiliated Hospital of Wannan Medical College (Yijishan Hospital of Wannan Medical College), Wuhu, Anhui, China; ^2^Takoradi Hospital, Takoradi, Western Region, Ghana; ^3^Department of Graduate School of Bengbu Medical University, Bengbu Medical University, Bengbu, Anhui, China; ^4^Department of Pulmonary and Critical Care Medicine, The Second People's Hospital of Wuhu, Wuhu, Anhui, China; ^5^Department of Cardiology, The First Affiliated Hospital of Wannan Medical College (Yijishan Hospital of Wannan Medical College), Wuhu, Anhui, China

**Keywords:** chronic obstructive pulmonary disease, burden of disease, epidemiology, Ghana, Africa, risk factors, Global Burden of Disease, COPD

## Abstract

**Objective:**

To assess the burden of chronic obstructive pulmonary disease (COPD) in Ghana within a global context, analyze temporal trends and risk factor attribution from 1990 to 2021, and project the future burden through 2050.

**Study design:**

Secondary analysis of Global Burden of Disease (GBD) 2021 data, using statistical modeling to evaluate trends in COPD prevalence, incidence, mortality, disability-adjusted life years (DALYs), and attributable risk factors. Future projections were generated using Bayesian Age-Period-Cohort (BAPC) modeling.

**Data source:**

GBD 2021 study, providing standardized estimates for 369 diseases across 204 countries and territories.

**Main outcome measures:**

COPD-related deaths, prevalence, incidence, DALYs, age-standardized rates (ASRs), risk factor attribution, percentage change, age-specific death rates, and projections to 2050.

**Results:**

From 1990 to 2021, Ghana experienced a 157% increase in COPD deaths (from 693 to 1,782), compared to a 49% global increase. Ghana's age-standardized death rate (ASDR) declined by only 7%, far below the global reduction of 37%. COPD prevalence in Ghana tripled, rising from 0.1 to 0.3 million, while incidence increased by 215% and DALYs by 171%. Globally, DALYs rose by 40% over the same period. In Ghana, household air pollution from solid fuel use accounted for 40% of COPD deaths, followed by ambient air pollution (25%). Globally, particulate matter pollution (41%) and smoking (36%) were dominant. Projections show continued increases in prevalence and incidence, particularly among adults aged 40–64, with plateauing DALYs and declining ASDR by the 2040s. Mortality increases sharply after age 60, with higher burden among males. Cohort analysis reveals rising mortality risk among those born after 1960.

**Conclusion:**

Despite modest ASDR reductions, Ghana's absolute COPD burden is increasing, driven by preventable risk factors such as household air pollution. These findings highlight the need for targeted interventions, including clean cook-stove programs, improved air quality monitoring, and expanded access to spirometry and early screening. Such efforts are essential to reduce COPD-related morbidity and mortality and advance Sustainable Development Goal (SDG) 3.4.

## 1 Introduction

Chronic obstructive pulmonary disease (COPD) represents a major global health concern in Ghana and worldwide (1). COPD is a progressive respiratory condition characterized by persistent airflow limitation and chronic airway inflammation, typically associated with long-term exposure to harmful particles or gases, such as tobacco smoke, indoor air pollution, and occupational dust (2). The disease imposes a substantial burden through impaired functional capacity, reduced quality of life, recurrent hospitalizations, and increased risk of premature mortality. According to the Global Burden of Disease (GBD) Study 2021, COPD ranked as the fourth leading cause of death globally, accounting for approximately 3.5 million deaths, equivalent to 5% of all deaths worldwide, affecting over 213 million individuals (3, 4).

Although COPD is widely recognized as a global health priority, its impact is disproportionately severe in LMICs, where more than 90% of COPD-related deaths occur (5). Despite the United Nations and World Health Organization introducing Sustainable Development Goal (SDG) Target 3.4 in 2015, which aims to reduce premature NCDs mortality by one-third by 2030, targeted strategies for COPD prevention and control still remain scarce in LMICs, including Ghana likely due to shared socioeconomic challenges and health system constrain (6, 7). In many such settings, the burden of COPD is compounded by environmental and occupational exposures, as well as health system weaknesses, such as fragmented surveillance, low clinical awareness, and limited policy prioritization (8, 9).

Ghana exemplifies many of the challenges in COPD detection and management. Despite the increasing prevalence of chronic respiratory conditions, COPD remains under-diagnosed, under-reported, and largely absent from the national non-communicable disease (NCD) policy framework (10). The Global Initiative for Chronic Obstructive Lung Disease (GOLD) provides standardized diagnostic and treatment guidelines; however, uptake in Ghana is limited by a shortage of trained personnel, insufficient access to diagnostic tools such as spirometry, and high out-of-pocket costs for medications (10, 11). Consequently, many patients are diagnosed only in advanced stages, often after irreversible lung damage has occurred (12).

In the global context, a population-based study in Central Asia (Almaty, Kazakhstan) using high-quality post-bronchodilator spirometry (FEV1/FVC below LLN) found that only 24% of confirmed COPD cases had ever been told they had the disease, while just 22% of prior physician diagnoses were confirmed by spirometry (13). These findings are consistent with reports emphasizing that COPD underdiagnosis is pervasive across both high- and low-income countries, driven by limited spirometry availability and inconsistent application of guidelines (14, 15). Such findings underscore a major diagnostic gap worldwide. Indeed, COPD prevalence estimates vary widely, with under-diagnosis ranging from 10 to 95% and over-diagnosis from 5 to 60%, driven by differences in diagnostic definitions and the limited availability of spirometry in rural areas of low- and middle- income countries where prevalence is likely high (16). Studies from similar sub-Saharan African countries provide illustrative insights, in Malawi, 22.5% of symptomatic adults were found to have COPD, yet only 4.6% had received a prior diagnosis (12). In Nigeria and Tunisia, similarly low diagnostic rates have been attributed to clinical inertia, poor guideline implementation, and inadequate access to pulmonary function testing (17, 18).

Beyond the clinical implications, COPD places a significant economic burden on households and health systems. The disease affects individuals during their most productive years, contributing to workforce attrition, increased dependency, and long-term socioeconomic vulnerability (19–21). In settings like Ghana, where health financing is heavily reliant on out-of-pocket expenditure, the cumulative costs of recurrent care, medication, and hospitalizations can be financially catastrophic (22–24). At the macroeconomic level, global COPD-related productivity losses are projected to exceed US$4.3 trillion by 2025, with LMICs bearing the greatest share of disability-adjusted life years (DALYs) lost (25, 26).

Despite the growing burden, COPD remains under-examined in Ghana, and nationally representative studies are scarce. While sub-Saharan Africa witnessed a 117% increase in age-standardized COPD prevalence between 1990 and 2019, Ghana's increase during the same period was estimated at nearly 194%, suggesting a potentially more severe national trajectory (5). However, most available estimates are derived from regional aggregates or extrapolated from global models, limiting their applicability for local health planning. The absence of longitudinal, country-specific assessments undermines Ghana's ability to track progress, allocate resources, and align with global targets such as Sustainable Development Goal (SDG) 3.4 (27).

This study seeks to fill this critical knowledge gap by analyzing long-term trends in COPD incidence, mortality, and burden in Ghana from 1990 to 2021, using data from the GBD 2021 study. In addition, we present projections through 2050 and situate Ghana's experience within regional and global contexts. By critically engaging with GBD estimates while acknowledging their limitations, we aim to provide actionable evidence for national policy development, health sector planning, and targeted interventions to mitigate the COPD burden.

## 2 Methods

### 2.1 Study design

This study employed a retrospective, population-based design to examine the burden of chronic obstructive pulmonary disease (COPD) in Ghana from 1990 to 2021, with projections extending to 2050. Secondary data from the Global Burden of Disease Study 2021 (GBD 2021) were used to quantify temporal trends in COPD incidence, prevalence, mortality, and disability-adjusted life years (DALYs), stratified by sex, age group, and calendar year. The analysis combined descriptive trend estimation, age-period-cohort (APC) decomposition modeling, and Bayesian forecasting techniques to assess both historical burden and future projections. To contextualize these quantitative findings, a targeted literature review was also conducted to situate results within Ghana's broader epidemiological and health systems landscape.

### 2.2 Data sources

Epidemiological estimates were obtained from the Institute for Health Metrics and Evaluation's (IHME) GBD 2021 dataset, accessed via the Global Health Data Exchange platform (https://vizhub.healthdata.org/gbd-results/). The GBD framework provides standardized annual estimates for 369 diseases and injuries across 204 countries and territories. COPD estimates are generated through systematic modeling of diverse data sources, including household surveys, health system records, and published literature. For Ghana, we extracted annual sex-specific and age-specific estimates for COPD incidence, prevalence, mortality, and DALYs, dis-aggregated by 5-year age bands and age-standardized rates, spanning 1990–2021. All estimates were reported with corresponding 95% uncertainty intervals (UIs) to reflect model-based variability.

To complement the GBD data, we conducted a supplementary literature review using PubMed and Google Scholar. The search strategy combined terms such as (“Chronic Obstructive Pulmonary Disease” OR “COPD”) AND (“Ghana” OR “Sub-Saharan Africa”) AND (“risk factors” OR “mortality” OR “prevalence” OR “DALYs”). We included peer-reviewed studies published between 1990 and 2021 that reported on COPD burden, risk exposures, or healthcare system barriers in Ghana or comparable low-resource African settings.

### 2.3 Trend analysis (1990–2021)

Temporal changes in COPD incidence, prevalence, mortality, and DALYs were analyzed using both absolute counts and age-standardized rates (ASRs) per 100,000 population. Percentage changes were calculated by comparing values from 1990 and 2021 using the formula: ((Value_2021 – Value_1990)/Value_1990) × 100.

These calculations were performed for both national and global estimates. Longitudinal trends and burden differentials were visualized using line graphs and temporal plots.

### 2.4 Age-Period-Cohort (APC) analysis

To disentangle temporal and generational effects underlying observed COPD trends, we employed an Age-Period-Cohort (APC) modeling framework. This approach captures the interplay of biological, environmental, and social factors influencing disease burden over time. The model estimates net drift (the overall annual percentage change across all age groups) and local drift (age-specific annual percentage changes). It distinguishes between age effects (α, reflecting biological susceptibility and developmental changes across the lifespan), period effects (β, reflecting external influences such as diagnostic availability, environmental exposures, or health policy shifts that simultaneously affect all age groups), and cohort effects (γ, representing generational differences shaped by unique historical exposures such as smoking prevalence or early-life air pollution). The model was specified within a generalized linear model (GLM) framework assuming a Poisson distribution for death counts, a log link function, and the log of the population at risk as an offset.

Mathematically, the Age-Period-Cohort (APC) model is represented as Y _ijk_ = μ + α _i_ + β _j_ + γ _k_ + ϵ _ijk_; where Y _ijk_ represents the observed disease burden for age group i, period j, and cohort k; is the overall intercept; and ϵ _ijk_ is the random error term (28).

### 2.5 Risk factor attribution

Attributable burden estimates for COPD were derived from the GBD 2021 Comparative Risk Assessment (CRA) framework, which quantifies disease outcomes linked to modifiable risk exposures. We assessed the fraction of COPD mortality and DALYs attributable to household air pollution from solid fuels, ambient particulate matter pollution, occupational exposures to gases, fumes, and dusts, tobacco smoking, secondhand smoke exposure, ambient ozone pollution, high temperature, and low temperature (5). We strictly followed the GBD 2021 CRA methodology. Population-attributable fractions (PAFs) were used, integrating exposure distributions, relative risks from meta-analyses, and theoretical minimum risk exposure levels (TMREL). Attributable fractions and burden estimates were extracted by year and sex from 1990 to 2021, with 95% UIs. For Ghana-specific projections, national-level PAFs were extrapolated using the BAPC model applied to historical GBD data.

### 2.6 Bayesian Age-Period-Cohort (BAPC) forecasting to 2050

Projections of COPD burden from 2022 to 2050 were generated using a Bayesian Age-Period-Cohort (BAPC) modeling framework. In this approach, age, period, and cohort effects were modeled as second- order random walks to ensure smooth temporal evolution. Gaussian priors were specified for the random walk terms, with precision parameters estimated from the data. Posterior distributions were approximated using Integrated Nested Laplace Approximation (INLA), which provides computational efficiency and avoids convergence issues common in Markov Chain Monte Carlo methods. The BAPC framework is particularly suited to large-scale epidemiological data because it can accommodate sparse or irregular time series while still producing stable estimates. By smoothing temporal effects and borrowing strength across adjacent age, period, and cohort groups, the model reduces random fluctuations and improves the reliability of long-term forecasts. The model incorporated historical COPD burden estimates from 1990 to 2021 together with demographic projections, thereby accounting for population growth, aging, and cohort-specific exposures. Forecasts were generated under the assumption that no major structural changes in risk factor distributions or healthcare interventions would occur beyond those already evident in the historical data. Outputs are presented with 95% uncertainty intervals (UIs) for GBD-derived indicators and 95% confidence intervals (CIs) for study-specific estimates, including age-standardized rates (ASRs). Analyses were implemented in R using the publicly available “BAPC” package (29).

### 2.7 Software and visualization

All data processing and graphical outputs were conducted using R and Microsoft Excel 2016. Line graphs and temporal plots were generated to illustrate observed and projected trends, APC decomposition results, and risk factor-specific burden estimates. All visualizations included 95% UIs to reflect the statistical variability inherent in modeled estimates.

### 2.8 Ethical considerations

This study relied exclusively on publicly available, de-identified secondary data obtained from the GBD 2021 repository and published literature. In accordance with ethical research guidelines, no primary data collection or human subject interaction was involved. As such, formal ethical review or institutional board approval was not required. All analyses complied with IHME's data use policies and adhered to best practices for responsible secondary data analysis.

## 3 Results

### 3.1 Mortality trends in Ghana and globally (1990–2021)

Between 1990 and 2021, the mortality burden of COPD in Ghana increased significantly by 157%, rising from 693 deaths (95% UI: 551–851) in 1990 to 1,782 deaths (95% UI: 1,402–2,231) in 2021. A similar upward trend was observed between 2019 and 2021. Despite the increase in absolute deaths, Ghana's age-standardized death rate (ASDR) declined modestly by 7.3%, from 14.94 to 13.84 per 100,000 (Table 1). Globally, COPD-related mortality rose substantially over the same period. The number of deaths increased by 49%, from 2.49 million (95% UI: 2.2–2.7 million) in 1990 to 3.7 million (95% UI: 3.3–4.0 million) in 2021, with a 3.4% rise between 2019 and 2021. However, the global ASDR showed a more pronounced decline of 37.1%, from 71.9 to 45.22 per 100,000 (Table 1).

**Table 1:** Global and Ghanaian Chronic Obstructive Pulmonary Disease (COPD) mortality, prevalence, incidence, and DALYs in 1990, 2019, and 2021.

**Location**	**Global**				**Ghana**			
**Epidemiological measure**	**1990**	**2019**	**2021**	**% Change (1990–2021)**	**1990**	**2019**	**2021**	**% change (1990–2021)**
**Deaths**								
Death cases (Count)	2,495,513 (2,238,986–2,694,755)	3,597,742 (3,274,671–3,877,018)	3,719,937 (3,347,912–4,084,218)	49.06%	693 (551–851)	1,766 (1,423–2,177)	1,782 (1,402–2,231)	157.13%
Death cases (Count) %Change	(1990–2019) 44.17%	(2019–2021) 3.40%	(1990–2021) 49.06%		(1990–2019) 154.90%	(2019–2021) 0.87%	(1990–2021) 157.13%	
Death cases (Rate)	46.79 (41.98–50.52)	46.45 (42.28–50.06)	47.14 (42.43–51.76)	0.75%	4.63 (3.68–5.68)	5.41 (4.36–6.67)	5.20 (4.09–6.51)	12.41%
Death cases (Rate) %Change	(1990–2019) −0.72%	(2019–2021) 1.48%	(1990–2021) 0.75%		(1990–2019) 16.98%	(2019–2021) −3.91%	(1990–2021) 12.41%	
ASDR (Rate)	71.92 (64.47–77.53)	46.09 (41.81–49.66)	45.22 (40.61–49.70)	−37.12%	14.94 (12.03–18.21)	14.26 (11.61–17.46)	13.85 (10.97–17.07)	−7.35%
ASDR %Change	(1990–2019) −35.91%	(2019–2021) −1.90%	(1990–2021) −37.12%		(1990–2019) −4.59%	(2019–2021) −2.89%	(1990–2021) −7.35%	
**Prevalence**								
Prevalent cases (Count)	100,544,855 (91,213,115–110,608,179)	204,278,957 (186,874,138–223,843,973)	213,387,446 (194,868,122–233,975,905)	112.23%	105,794 (93,384–118,884)	297,944 (261,710–333,646)	320,572 (285,498–355,227)	203.01%
Prevalent cases (Count) %Change	(1990–2019) 103.17%	(2019–2021) 4.46%	(1990–2021) 112.23%		(1990–2019) 181.63%	(2019–2021) 7.59%	(1990–2021) 203.01%	
Prevalent cases (Rate)	1,885.11 (1,710.15–2,073.79)	2,637.45 (2,412.73–2,890.05)	2,704.07 (2,469.39–2,964.97)	43.44%	706.64 (623.74–794.06)	913.32 (802.24–1,022.76)	936.09 (833.67–1,037.28)	32.47%
Prevalent cases (Rate) %Change	(1990–2019) 39.91%	(2019–2021) 2.53%	(1990–2021) 43.44%		(1990–2019) 29.25%	(2019–2021) 2.49%	(1990–2021)	32.47%
ASPR	2,550.02 (2,318.34–2,806.32)	2,520.67 (2,306.51–2,756.83)	2,512.86 (2,293.93–2,748.52)	−1.46%	1,438.67 (1,260.06–1,614.25)	1,639.86 (1,438.25–1,850.37)	1,672.26 (1,471.40–1,876.37)	16.24%
ASPR %Change	(1990–2019) −1.15%	(2019–2021) −0.31%	(1990–2021) −1.46%		(1990–2019) 13.98%	(2019–2021) 1.98%	(1990–2021) 16.24%	
**Incidence**								
Incidence cases (Count)	8,054,865 (7,419,999–8,706,820)	16,126,485 (14,787,210–17,514,355)	16,895,445 (15,471,347–18,335,691)	109.75%	6,789 (5,978–7,582)	19,820 (17,528–22,278)	21,374 (18,908–23,900)	214.85%
Incidence Cases (Count) %Change	(1990–2019) 100.21%	(2019–2021) 4.77%	(1990–2021) 109.75%		(1990–2019) 191.95%	(2019–2021) 7.84%	(1990–2021) 214.85%	
Incidence cases (Rate)	151.02 (139.12–163.24)	208.21 (190.92–226.13)	214.10 (196.05–232.35)	41.77%	45.34 (39.93–50.64)	60.76 (53.73–68.29)	62.41 (55.21–69.79)	37.64%
**Epidemiological measure**	**1990**	**2019**	**2021**	**% Change (1990–2021)**	**1990**	**2019**	**2021**	**% change (1990–2021)**
Incidence cases (Rate) %Change	(1990–2019) 37.87%	(2019–2021) 2.83%	(1990–2021) 41.77%		(1990–2019) 33.99%	(2019–2021) 2.73%	(1990–2021) 37.64%	
ASIR	202.43 (186.70–218.25)	197.30 (181.85–213.09)	197.37 (181.65–213.42)	−2.50%	97.41 (86.68–108.56)	112.31 (99.09–126.58)	114.47 (100.93–129.86)	17.51%
ASIR %Change	(1990–2019) −2.54%	(2019–2021) 0.04%	(1990–2021) −2.50%		(1990–2019) 15.30%	(2019–2021) 1.92%	(1990–2021) 17.51%	
**DALYs**								
DALYs cases (Count)	56,857,290 (51,293,861–61,412,142)	77,327,992 (71,819,023–82,519,473)	79,779,695 (74,026,373–86,011,406)	40.32%	26,215 (22,184–30,788)	68,625 (58,617–80,690)	71,052 (59,466–83,838)	171.04%
DALYs cases (Count) %Change	(1990–2019) 36.00%	(2019–2021) 3.17%	(1990–2021) 40.32%		(1990–2019) 161.78%	(2019–2021) 3.54%	(1990–2021)	171.04%
DALYs cases (Rate)	1,066.02 (961.71–1,151.42)	998.38 (927.26–1,065.41)	1,010.98 (938.07–1,089.94)	−5.16%	175.10 (148.17–205.64)	210.36 (179.68–247.35)	207.48 (173.64–244.81)	18.49%
DALYs cases (Rate) %Change	(1990–2019) −6.34%	(2019–2021) 1.26%	(1990–2021) −5.16%		(1990–2019) 20.14%	(2019–2021) 1.37%	(1990–2021) 18.49%	
ASDALYs (Rate)	1,492.64 (1,342.46–1,609.30)	958.62 (889.80–1,023.31)	940.66 (871.48–1,014.59)	−36.98%	410.50 (348.32–481.99)	419.26 (358.29–488.14)	411.82 (344.86–486.00)	0.32%
ASDALYs (Rate) %Change	(1990–2019) −35.78%	(2019–2021) −1.87%	(1990–2021) −36.98%		(1990–2019) 2.13%	(2019–2021) −1.77%	(1990–2021) 0.32%	

This table presents the number and age-standardized rates (per 100,000 population) for deaths, prevalence, incidence, and disability-adjusted life years (DALYs) due to COPD, along with the percentage change in rates from 1990 to 2021. Values in parentheses indicate 95% uncertainty intervals (UIs). Data are soured from the Global Burden of Disease (GBD) 2021 Study.

COPD, chronic obstructive pulmonary disease; DALYs, disability-adjusted life years; ASDR, age-standardized death rate; ASPR, age-standardized prevalence rate; ASIR, age-standardized incidence rate; ASDALYs, age-standardized DALYs; UI, uncertainty interval, %, percentage.

### 3.2 Prevalence trends in Ghana and globally (1990–2021)

In Ghana, the number of prevalent COPD cases increased by 203%, from 0.1 million (95% UI: 0.093–0.118 million) in 1990 to 0.3 million (95% UI: 0.285–0.355 million) in 2021. Between 2019 and 2021 alone, prevalence rose by 7.6%. The age-standardized prevalence rate (ASPR) also rose by 16.2%, from 1,438.7 (95% UI: 1,260.1–1,614.2) to 1,672.3 (95% UI: 1,471.4–1,876.4) per 100,000 (Table 1). Globally, the number of prevalent COPD cases more than doubled, increasing by 112% from 10 million (95% UI: 9.1–11.1 million) in 1990 to 21 million (95% UI: 19.5–23.4 million) in 2021, with a 4.45% increase between 2019 and 2021. In contrast to Ghana, the global ASPR declined slightly by 6.9%, from 2,550.0 (95% UI: 2,318.3–2,806.3) to 2,512.9 (95% UI: 2,293.9–2,748.5) per 100,000 (Table 1).

### 3.3 Incidence trends in Ghana and globally (1990–2021)

Ghana recorded a 215% increase in incident COPD cases between 1990 and 2021, with the number of new cases rising from 6,789 (95% UI: 5,978–7,582) to 21,374 (95% UI: 18,900–23,899). The age-standardized incidence rate (ASIR) increased by 17.5%, from 97.4 to 114.5 per 100,000 during the same period (Table 1). Globally, incident COPD cases also doubled, increasing by 110% from 8.05 million (95% UI: 7.4–8.7 million) in 1990 to 16.89 million (95% UI: 15.5–18.3 million) in 2021. However, the global ASIR declined slightly by 2.5%, from 202.4 to 197.4 per 100,000 (Table 1).

### 3.4 Trends in DALYs in Ghana and globally (1990–2021)

In Ghana, DALYs attributed to COPD rose sharply by 171%, from 26,215 (95% UI: 22,183–30,780) in 1990 to 71,052 (95% UI: 59,466–83,838) in 2021. The age-standardized DALY rate (ASDALYs) remained essentially stable, with only a 0.3% change from 419.2 to 411.8 per 100,000 (Table 1). Globally, DALYs due to COPD increased by 40.3%, from 56.85 million (95% UI: 52.3–62.4 million) in 1990 to 79.77 million (95% UI: 74.0–86.0 million) in 2021. In contrast to Ghana, the global ASDALYs declined by 37.0%, from 1,492.6 to 958.6 per 100,000 (Table 1).

### 3.5 Attributable risk factors in Ghana and globally

**Figure 1:**
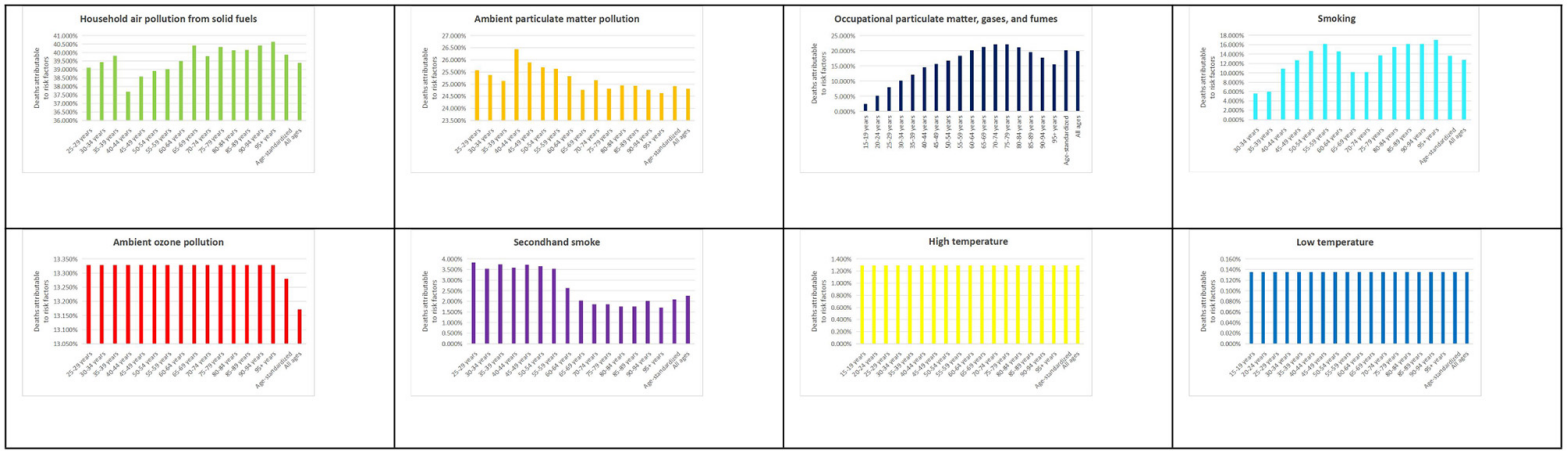
Percentage of deaths from chronic obstructive pulmonary disease (COPD) attributable to all risk factors in Ghana in 2021. This figure presents the proportion of COPD-related deaths attributable to various risk factors, based on data from the Global Burden of Disease (GBD) 2021 Study (https://vizhub.healthdata.org/gbd-results/).

In Ghana, household air pollution from solid fuels emerged as the leading risk factor, accounting for 40% of COPD-related deaths. This was followed by ambient particulate matter pollution (25%), occupational exposures (20%), and smoking (15%). Additional contributors included ambient ozone pollution (15%), secondhand smoke (1.5%), and extreme temperatures (0.2% each; Figure 1). Globally, ambient particulate matter pollution was the dominant risk factor for COPD mortality (40.95%), followed by smoking (35.64%) and household air pollution (18.5%). High temperatures contributed the least (Supplementary Figure S1). For DALYs, Ghana's pattern mirrored that of mortality: household air pollution accounted for 39.2%, ambient particulate matter for 24.4%, occupational exposures for 19%, and smoking for 11.9% (Supplementary Figures S2, S3). This profile differs from global trends, where smoking plays a comparatively larger role (Supplementary Figures S4, S5), emphasizing Ghana's environmental and occupational risk profile.

### 3.6 Bayesian Age-Period-Cohort (BAPC) trend of COPD mortality in Ghana (1990–2021)

**Figure 2:**
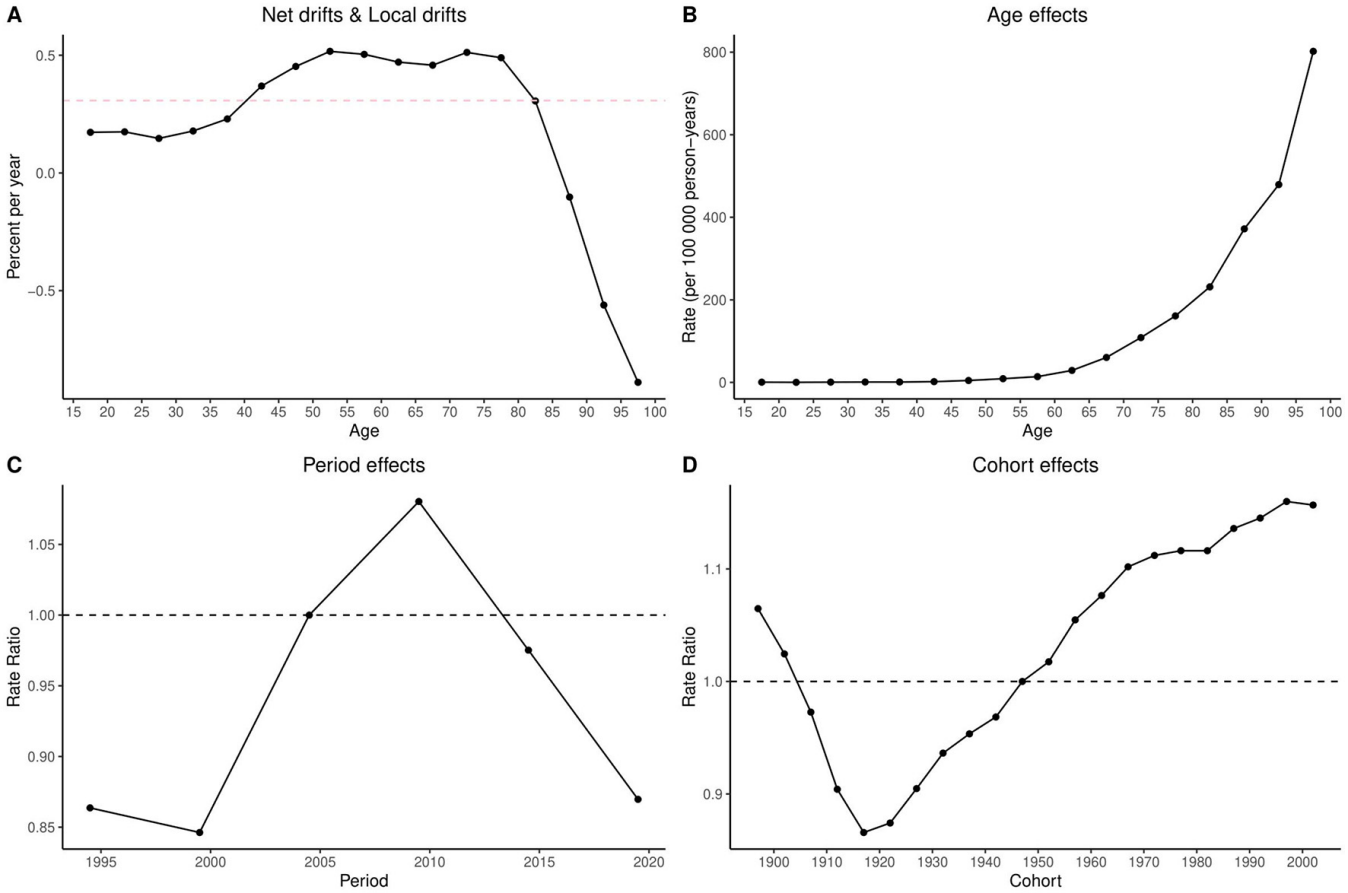
Age-Period-Cohort (APC) analysis of COPD mortality in Ghana (1990–2021): net drifts, local drifts, age, period, and cohort effects. The figure comprises four panels: **(A)** Net drifts and local drifts, showing overall and age-specific annual percent changes in mortality rates; **(B)** Age effects, depicting mortality rates per 100,000 person-years across age groups; **(C)** Period effects, illustrating rate ratios of mortality across calendar years (1990–2021); and **(D)** Cohort effects, showing rate ratios of mortality by birth cohort (1900–2000). This analysis reveals both temporal trends and generational variations in COPD mortality risk in Ghana. APC, age-period-cohort analysis; COPD, chronic obstructive pulmonary disease; net drift, overall annual percent change in age-adjusted mortality rates; local drift, age-specific annual percent change in mortality rates; rate ratio, the relative risk of mortality compared to a reference period or cohort.

The Bayesian Age-Period-Cohort (BAPC) analysis for Ghana revealed a marked temporal trend across age, period, and cohort effects. Net drift estimates indicated an overall decline in annual mortality rates, ranging from approximately 0% at age 15 to −2% at age 100. Notable reductions were observed in individuals aged 85 and above. Age effects showed mortality beginning to increase at age 65, peaking at age 90 and above. Period effects indicated a modest reduction in mortality before 2005, followed by a peak around 2010 and a steep decline through 2020. Cohort effects were stable or declining for those born before 1950, with rising death rates among cohorts born after 1960 (Figure 2).

### 3.7 Patterns of Age- and Sex-Specific COPD mortality in Ghana, 2021

**Figure 3:**
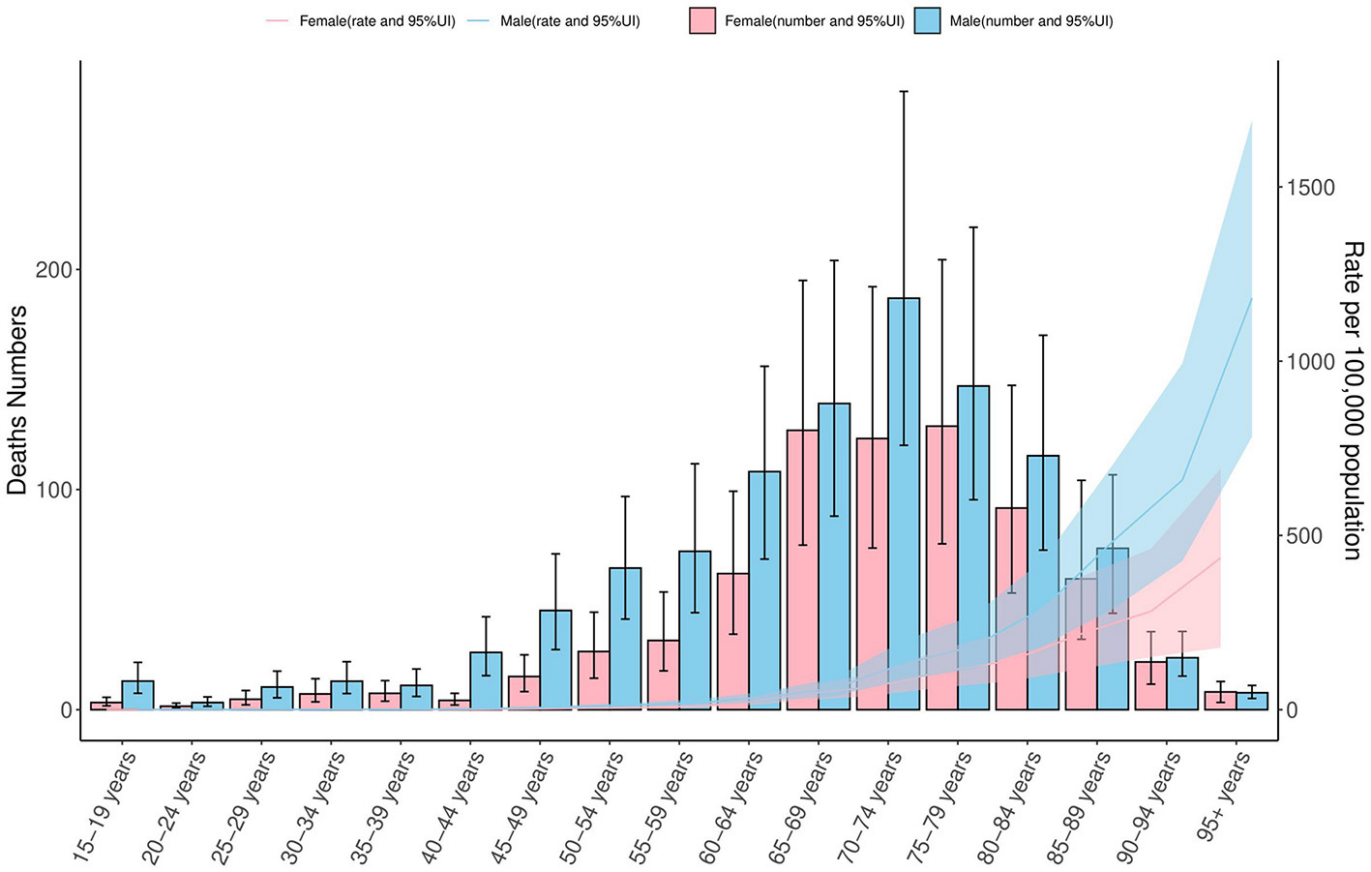
COPD mortality by age and sex in Ghana, 2021: counts and rates per 100,000 population. This figure presents age-specific mortality from COPD in Ghana for the year 2021, stratified by sex. The left y-axis displays the number of deaths (ranging from 0 to 200), while the right y-axis shows the corresponding mortality rates per 100,000 population (ranging from 0 to 1,500). Bars with 95% confidence intervals (CIs) illustrate death counts for females (pink) and males (blue), and shaded areas represent the corresponding age-specific mortality rates. The figure highlights sex-based and age-related differences in COPD mortality, based on data from the Global Burden of Disease (GBD) 2021 Study (https://vizhub.healthdata.org/gbd-results/). COPD, chronic obstructive pulmonary disease; CI, confidence interval; rate per 100,000 population, a standardized metric used to compare mortality across age groups and population sizes.

In 2021, COPD mortality in Ghana demonstrated a clear age-dependent pattern for both sexes. Deaths remained below 50 between ages 15–44, increased gradually from age 45–49, and rose sharply from age 60 onwards. The highest mortality count was observed in males aged 70–74 (approximately 200 deaths) and in females aged 75–79 (approximately 150 deaths). Age-specific mortality rates were low before age 50–54 but rose significantly thereafter, peaking at approximately at 1,200 per 100,000 among males aged 95+, and 500 per 100,000 among females of the same age group. Across all age groups, males consistently exhibited higher mortality rates. Wider 95% uncertainty intervals in older age groups highlight the estimation variability inherent in GBD 2021 data (Figure 3).

### 3.8 Ghana's projections to 2050

**Figure 4:**
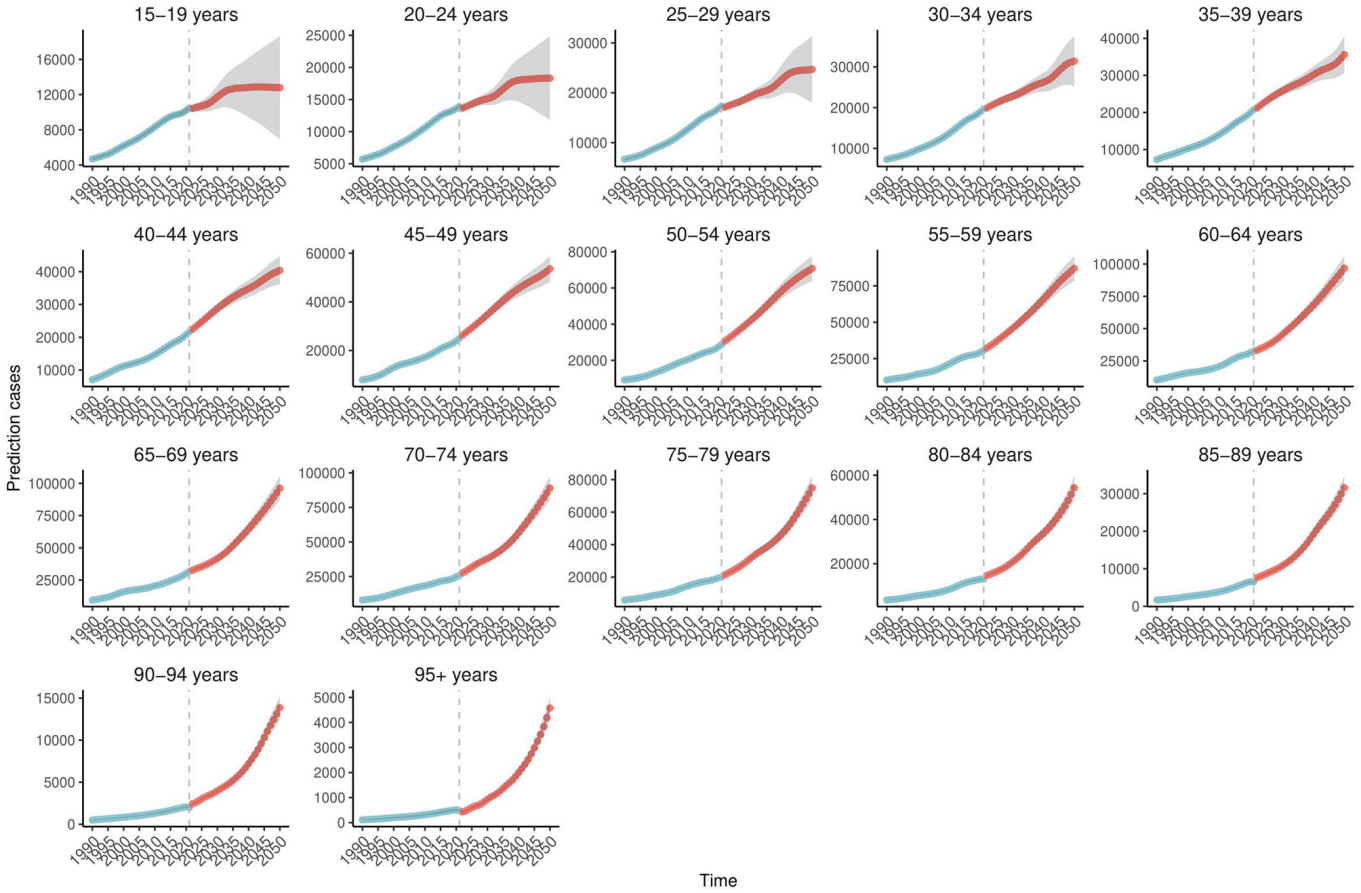
Age-specific trends in projected COPD prevalence in Ghana, 1990–2050. This figure presents age-specific projections of COPD prevalence in Ghana from 1990 to 2050, based on Bayesian forecasting models. The y-axis indicates the number of cases. The projections reveal a substantial increase among adults aged 40–64 years, with consistently lower rates observed in younger age groups (15–24 years). Shaded areas represent uncertainty intervals, highlighting differences in projected trends across age categories. Data are derived from the Global Burden of Disease (GBD) 2021 Study (https://vizhub.healthdata.org/gbd-results/). COPD, chronic obstructive pulmonary disease; Bayesian forecasting models, statistical models that apply Bayesian inference to predict future trends based on historical data and associated uncertainty.

**Figure 5:**
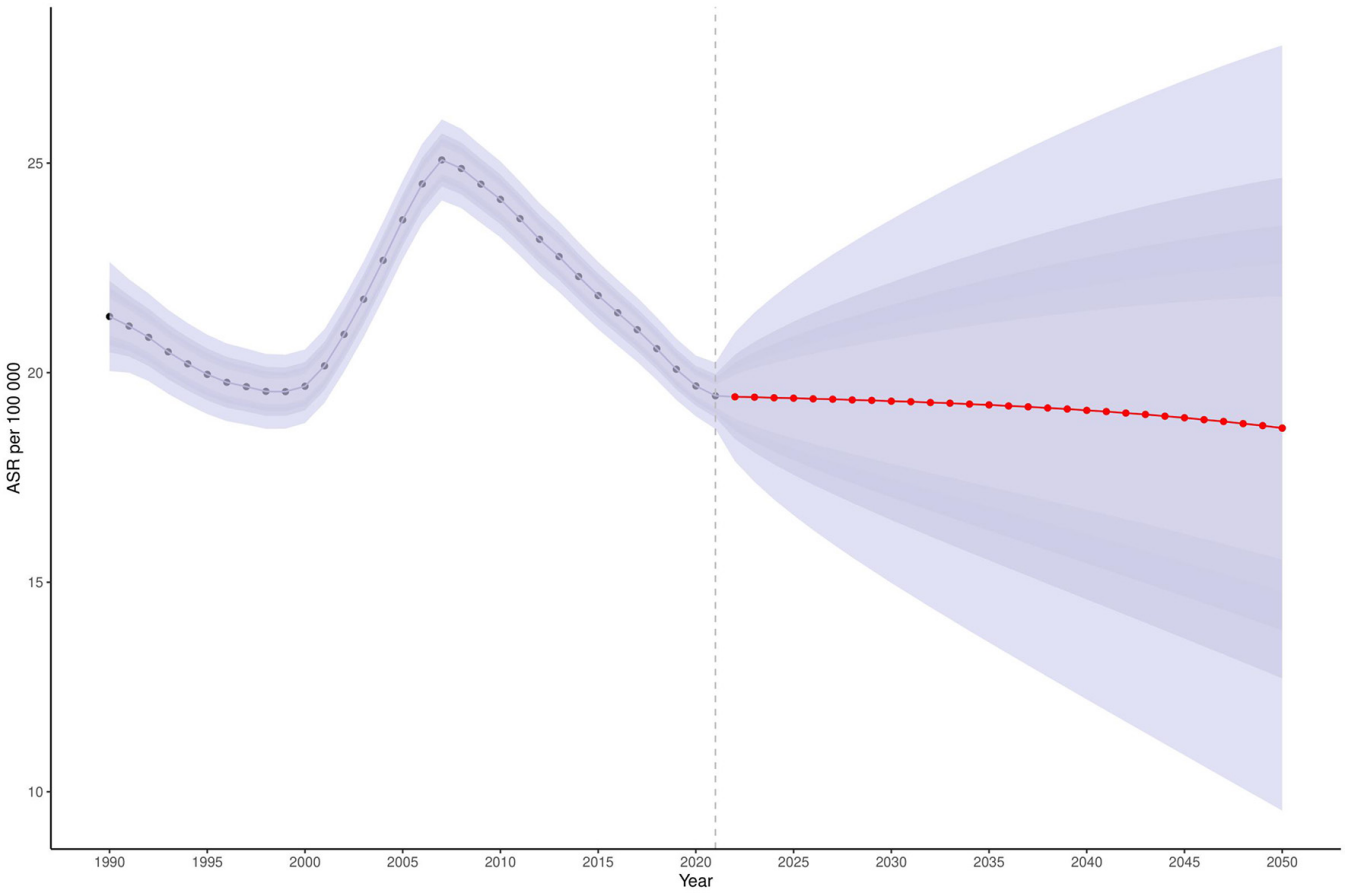
Projected ASDR for COPD in Ghana, 2025–2050. This figure presents the projected age-standardized death rate (ASDR) for COPD in Ghana between 2025 and 2050, using Bayesian forecasting models. The y-axis represents the ASDR per 100,000 population. A marked decline is projected beginning in the 2040s and continuing into the 2050s. Shaded areas indicate uncertainty intervals around the projected estimates. Data are sourced from the Global Burden of Disease (GBD) 2021 Study (https://vizhub.healthdata.org/gbd-results/). COPD, chronic obstructive pulmonary disease; ASDR, age-standardized death rate; Bayesian forecasting models, statistical models using Bayesian inference to estimate future outcomes based on historical data.

Bayesian forecasting predicts a sustained increase in Ghana's age-standardized prevalence (ASPR) and incidence rates (ASIR) of COPD through 2050 (Supplementary Figures S6, S7), particularly among adults aged 40–64 years (Figure 4). In contrast, the burden is expected to remain relatively low among individuals aged 15–24 years (Supplementary Figures S8, S9). Encouragingly, the ASDR is projected to decline significantly during the 2040s and 2050s (Figure 5). ASDALYs are also expected to gradually decline (Supplementary Figure S10), although age-specific DALY counts will rise sharply from age 64, peaking between ages 85 and 89 (Supplementary Figure S11).

## 4 Discussion

We systematically analyzed the burden of chronic obstructive pulmonary disease (COPD) in Ghana and globally from 1990 to 2021, with projections to 2050, using data from the Global Burden of Disease Study 2021. Unlike the global trend of declining COPD mortality, Ghana has shown relatively stable or rising death rates since 2010. Since 1990, COPD incidence in Ghana has increased by 215%, compared to a global rise of 110%, with projections indicating further growth in line with population aging and expansion. Underdiagnosis remains a major concern, with detection rates estimated to be low in Ghana and other low- and middle-income countries. Ghana's DALYs from COPD have also increased, which may be influenced by delayed diagnosis, limited access to medications, and Comorbidities such as tuberculosis. Household air pollution from biomass fuel use continues to be a leading risk factor, alongside ambient particulate matter and occupational exposures. Socioeconomic impacts include potential effects on poverty and labor productivity, reflecting the broader consequences of chronic disease. Projections to 2050 suggest continued increases in COPD prevalence, particularly among adults aged 40–64, and rising DALYs in older populations. These finding highlight the relevance of strengthening diagnostic tools, including wider availability and use of spirometry, to improve early detection and management of COPD in Ghana.

### 4.1 Mortality, prevalence, incidence and DALYs in trends in Ghana in global context

While global COPD mortality has generally declined, Ghana's age-standardized death rate (ASDR) appears to have stagnated or slightly increased since 2010, placing it among the countries reporting rising non-communicable disease mortality (27). This pattern is broadly consistent with the period effects identified in our BAPC analysis, which suggested a mortality peak around 2010. Net and local drift estimates point to modest declines in certain age groups, particularly men aged 70–74 and women aged 75–79, but overall burden levels across incidence, prevalence, mortality, and DALYs remained relatively stable between 2019 and 2021 (Table 1). COPD prevalence in Ghana has increased steadily over the past three decades, particularly among older adults, a trend that mirrors global and regional patterns (17, 30). Although sub-Saharan Africa currently reports the lowest global prevalence, projections suggest the region may experience the largest absolute increase in COPD cases by 2050 (31, 32). Ghana's population is expected to grow from 33.78 million in 2023 to 50.55 million by 2050, which could amplify the impact of this trend (33).

Accurate confirmation of COPD requires high-quality post-bronchodilator spirometry to demonstrate persistent airflow limitation (2, 15). In Ghana, spirometry is performed in tertiary hospitals and has been used in peri-urban hospital research and hospital audits documenting substantial obstructive patterns (25.5% obstructive in a Korle Bu teaching-hospital lung function audit) (34, 35). The EUAA (2023) topical report on pulmonology in Ghana records a public tertiary hospital fee for lung-function testing of approximately GHS 59.41, illustrating that the unit test cost can be modest where the service exists (36); however, there are no published data that quantify spirometry coverage across district and primary-care facilities in Ghana. Pan-African commentaries and multi-country reviews identify recurrent barriers to scaling spirometry, limited trained personnel, shortages of consumables (mouthpieces/filters), calibration and maintenance challenges, and inadequate clinician training in interpretation, which together perpetuate underdiagnosis and late presentation of COPD in many African countries (37). Since 1990, Ghana's COPD incidence has risen by an estimated 215%, compared with a global increase of 110%. Taken together, these findings highlight the likely importance of strengthening diagnostic capacity and promoting earlier detection to more effectively address Ghana's growing COPD burden.

DALYs attributable to COPD in Ghana have risen sharply, with patterns that may be influenced by premature mortality and disability linked to limited access to essential medications such as salbutamol and ipratropium bromide (38). Access to essential COPD medicines in Ghana remains limited and uneven across levels of care. In 2014, only 39.1% of healthcare facilities reported access to inhaled short-acting β_2_-agonists (SABA), with availability at 44.0% of health centers and 100% at district and regional hospitals. Access to inhaled corticosteroids (ICS) was lower, at 17.4% overall, with no availability at primary care facilities or health centers, 67.0% at district hospitals, and 100% at regional hospitals (39). Although these data are a decade old, they indicate persistent gaps in equitable access, particularly at the primary care level where most patients first seek treatment. Addressing these gaps could support earlier management and improve continuity of care. Ghana's increasing life expectancy, from 55 years in 1990 to 66 years in 2021, may also contribute to the COPD burden by increasing the likelihood of late-onset disease (33). Our BAPC analysis supports this, showing a rise in COPD mortality after age 65 in both sexes. These demographic shifts highlight the importance of age- and sex-sensitive public health planning, including equitable chronic disease management across regions.

One factor that may contribute to the high COPD DALYs in Ghana is the role of Comorbidities, particularly tuberculosis. Prior tuberculosis has been consistently associated with increased COPD risk, and this relationship is relevant in Ghana, where TB remains the second leading infectious cause of mortality (22, 33, 40). Although Ghana-specific data on TB-COPD overlap are limited, the high national TB burden indicates that post-TB lung disease could be an under-recognized component of COPD morbidity and mortality. Incorporating post-TB lung health into diagnostic strategies in TB clinics, alongside improved access to respiratory care, would help clarify its contribution to COPD DALYs.

### 4.2 Attributable risk factors

Household air pollution remains the leading COPD risk factor in Ghana, largely due to continued reliance on biomass fuels such as wood and charcoal. Surveys indicate that 70%−80% of households have historically relied on solid fuels, with rural dependence remaining high despite rising LPG adoption in urban areas (41). This suggests that large populations remain chronically exposed to smoke in domestic settings. In Ghana, pregnant women and children are exposed to biomass smoke have been found to exhibit elevated respiratory symptoms (42). Personal-monitoring studies quantify the magnitude of these exposures. Twenty-four hour personal PM_2_.5 concentrations among rural Ghanaian primary cooks averaging 128.5 μg/m3, with concurrent kitchen measurements reaching 446.8 μg/m3, more than eightfold and 30-fold higher, respectively, than the WHO 2021 24-h PM_2_.5 guideline of 15 μg/m3 (43, 44). These peaks occurred during cooking and dominated daily exposure profiles. While these personal exposure levels are driven primarily by cooking activity, emerging evidence suggests that ambient air pollution also plays a significant role in shaping indoor PM_2_.5 concentrations, particularly in settings with high outdoor pollution.

The CLEAN Air (Africa) study in peri-urban Obuasi found that charcoal and LPG users had similar kitchen (56 vs. 52 μg/m3) and personal (60 vs. 49 μg/m3) PM_2_.5 exposures, unlike other sites where LPG use showed clear reductions (45). The authors attributed this to high ambient PM_2_.5 levels limiting the benefits of fuel switching through outdoor-to-indoor infiltration. Similar dynamics have been observed in high-pollution settings such as Kazakhstan, where up to 40% of ambient PM_2_.5 concentrations were found to penetrate indoors even with windows closed, and higher levels when doors were opened (46). WHO city data for Accra reported mean residential PM_2_.5 49.5 μg/m3 in 2014–2015, with satellite-based regional estimates around 36 μg/m3. Micro-environmental modeling in Accra indicates that time-activity patterns, kitchen configuration, ventilation, and outdoor infiltration together influence personal PM_2_.5 dose, and that paired indoor–outdoor monitoring can help resolve setting-specific exposure drivers (47). In line with these exposure patterns, ambient particulate matter pollution has been identified as a major contributor to COPD burden in Ghana. A recent analysis estimated that ambient PM_2_.5 exposure was responsible for more than 7,300 deaths in 2021, with 56% attributable to biomass burning and 9.6% to fossil fuel combustion (48). Taken together with household exposure data, these findings illustrate the dual and interacting contributions of household and ambient sources to respiratory health risks. This combined evidence supports the interpretation that household air pollution, particularly when compounded by high ambient levels, is a proximate determinant of Ghana's rising COPD burden and helps explain why prevalence and DALYs have continued to increase despite some transitions to cleaner fuels.

In addition to household and ambient sources of particulate pollution, occupational inhalation hazards contribute to Ghana's COPD burden. Occupational exposures rank as Ghana's third-leading COPD risk factor, with studies documenting high levels of vapors, gases, dust, and fumes (VGDF) in key sectors. In 2023, approximately 35% of Ghana's workforce was engaged in agriculture, and 80% participated in informal employment across all sectors, including agriculture, based on World Bank and ILO estimates (49, 50). These sectors, including agricultural burning, fish smoking, small-scale mining, sawmilling, and construction, expose a substantial portion of the workforce to respiratory hazards.

Measured exposure studies highlight these risks. Among coastal fish smokers using traditional “Chorkor” ovens, mean indoor PM_2_.5 concentrations of 322 μg/m3 (range 164–493) were reported, approximately 21 times higher than WHO's 24-h guideline of 15 μg/m3, alongside elevated indoor VOC levels (8.6 vs. 4.4 mg/m3 at control sites) (51). A complementary study documented mean 6-h CO exposures of 18 ppm among Ghanaian fish smokers (52). Underground gold miners in formal operations were exposed to respirable dust levels up to 0.221 mg/m3 (e.g., shotcrete operators) and crystalline silica, with 41% exceeding the NIOSH permissible exposure limit (PEL) of 0.05 mg/m3, and 49% exceeding the Mine Safety and Health Administration (MSHA) diesel particulate matter (DPM) PEL of 160 μg/m3 (53). Informal e-waste recyclers at Agbogbloshie recorded median PM_2_.5 exposures of 99 ± 56 μg/m3, nearly double the levels at fixed-site monitors, and elevated polycyclic aromatic hydrocarbons (PAHs), with levels 10%−25% higher than a nearby community (54). Sawmill workers in Cape Coast faced inhalable wood dust levels up to 6.55 mg/m3 (mean: 3.09 mg/m3), exceeding the NIOSH threshold limit of 1 mg/m3 for hardwoods, and experienced acute lung function declines, including reductions in forced vital capacity (FVC) from 3.62 to 3.49 L and forced expiratory volume (FEV1) from 3.11 to 3.00 L after work shifts (55). Although nationally representative personal-monitor data on VGDF prevalence are lacking, these sector-specific studies, combined with workforce shares, indicate a substantial occupational contribution to Ghana's COPD burden. Literature from similar contexts suggests that measures such as national occupational exposure surveillance, enforcement of exposure limits, provision of personal protective equipment, and adoption of cleaner processing technologies could help reduce risks in high-exposure sectors.

Tobacco smoking is the leading COPD risk factor globally (56), but in Ghana it ranks fourth, a pattern that may reflect the impact of strong anti-tobacco legislation, sustained public health campaigns, and prevailing cultural disapproval of smoking (57–60). Recent reports, however, indicate a growing popularity of shisha (flavored tobacco mixture that is smoked using a hookah or a water pipe) among urban youth, which could increase future COPD risk if left unaddressed (61). Evidence from other settings suggests that targeted measures, such as school-based awareness programs, youth-focused cessation services, and enforcement of existing restrictions on flavored tobacco products, could help curb this emerging trend (62, 63).

### 4.3 Projections of COPD burden in Ghana to 2050

Bayesian forecasting indicates that COPD prevalence in Ghana will continue rising through 2050, particularly among adults aged 40–64. This trend is consistent with the country's increasing life expectancy (from 55.65 years in 1990 to 66 years in 2021) and the cumulative impact of early-life exposures. Incidence is expected to climb sharply in middle-aged adults, while younger populations (15–24) remain vulnerable. Although age-specific mortality is projected to decline, the total number of COPD deaths will increase in the 2040s and 2050s due to population aging. DALYs are expected to plateau in terms of age-standardized rates but rise in absolute numbers, especially from age 64, peaking at 85–89 years. These projections suggest that early-stage interventions could be valuable, such as targeted screening for high-risk occupational groups (e.g., fish smokers, artisanal miners) and adolescents with known exposure histories, alongside health-education programs. Preparing for the needs of an aging population will also require investment in geriatric respiratory-care infrastructure, including trained personnel and regionally distributed services.

### 4.4 Policy recommendations

Addressing Ghana's COPD burden requires coordinated, context-specific action. (1) Household energy transition should be prioritized by promoting clean cookstoves and LPG adoption through targeted subsidies for low-income households, microloan schemes for rural women's cooperatives, and tax relief on LPG cylinders and stoves, with uptake systematically monitored in high biomass-use districts. (2) Diagnostic capacity can be strengthened by expanding access to spirometry, particularly through integration of portable devices into district hospitals and TB clinics, supported by structured training and certification programs for respiratory nurses and technicians, with results linked to NHIS reimbursement to reduce out-of-pocket costs. (3) Targeted screening initiatives should be implemented for high-risk groups, including older adults, individuals with prior TB, and women with prolonged biomass exposure, to facilitate early detection and reduce under-diagnosis. (4) Occupational protection requires enforcing dust and chemical exposure standards in mining, saw-milling, and fish-smoking sectors, while scaling up improved “Chorkor” ovens, strengthening occupational health surveillance, and expanding access to affordable PPE, particularly in informal employment settings. (5) Environmental regulation must be reinforced through stricter enforcement of policies addressing traffic emissions, industrial pollution, and open waste burning, with compliance data collected and publicly reported for major cities such as Accra and Kumasi. (6) Finally, policy integration is essential: clean household energy and air quality targets should be embedded within Ghana's Renewable Energy Master Plan and aligned with WHO NCD frameworks, while partnerships with local institutions and civil society can help pilot portable indoor air monitors and ensure coherence between health and energy policies. Together, these context-specific and actionable measures provide a practical road-map for mitigating COPD risk, enhancing early detection, and reducing future disease burden in Ghana.

### 4.5 Limitations of this study

This study has several constraints that should be considered when interpreting the findings. (1) Reliance on GBD 2021 estimates introduces uncertainty, as data for Ghana are sparse and often extrapolated from regional or global patterns, which may underestimate the true burden. In particular, the Comparative Risk Assessment (CRA) framework depends on modeled exposure distributions that may underrepresent rural populations, relative risk estimates derived largely from non-African cohorts, and the assumption of uniform exposure–response relationships across settings. These factors may introduce uncertainty into Ghana-specific risk attribution, though the CRA framework remains the most widely validated approach for global comparative analysis. (2) The absence of subnational granularity likely masks disparities across Ghana's 16 regions, limiting the ability to design region-specific interventions. (3) Systemic underdiagnosis, driven by limited access to spirometry and low awareness among patients and providers, further reduces accuracy. (4) Nationally representative data on occupational exposures are not yet available, with current evidence drawn from small sector-specific studies that cannot be generalized to the entire workforce. (5) Finally, projections assume the continuation of past trends, which may not fully capture the effects of new policies, technologies, or interventions introduced in the future.

### 4.6 Future research direction

To strengthen the evidence base and guide policy, future work should prioritize: (1) generating region- and sex- specific COPD prevalence data to uncover subnational disparities masked by national averages; (2) quantifying the contribution of comorbidities such as tuberculosis and recurrent respiratory infections to COPD progression; (3) assessing the availability and affordability of diagnostic tools, particularly spirometry and bronchoscopy, in rural and peri-urban areas; (4) evaluating adherence to GOLD guidelines, including provider training in spirometry interpretation and pulmonary rehabilitation; (5) investigating undiagnosed COPD among younger populations exposed to biomass smoke and urban air pollution; (6) conducting longitudinal cohort studies to assess the long-term effectiveness and cost-efficiency of interventions; and (7) fostering community-engaged research aligned with SDG 3.4 to ensure interventions are context-appropriate and widely adopted.

## 5 Conclusions

Chronic Obstructive Pulmonary Disease (COPD) remains an important public health concern globally, and Ghana is experiencing a rising burden. Unlike global patterns where tobacco use predominates, Ghana's COPD profile is influenced largely by household air pollution from biomass fuel use, together with ambient air pollution and occupational exposures in sectors such as fish smoking, mining, and sawmilling. Projections through 2050 suggest that prevalence and mortality may increase, driven by population aging and early-life exposures, even as age-specific mortality shows signs of modest decline. Addressing Ghana's diagnostic gap is an important step in responding to this trajectory. Spirometry, the gold standard for COPD confirmation, is currently concentrated in tertiary hospitals, limiting timely diagnosis and likely underestimating the true burden. Expanding access to portable, affordable spirometers in district hospitals and TB clinics, alongside training and sustainable financing through the National Health Insurance Scheme, may improve early detection. Gaps in the availability of essential inhaled medicines also point to the need for stronger supply chains and more equitable access across all levels of care. Policy measures could include scaling up clean cook-stove and LPG adoption, strengthening enforcement of environmental and occupational exposure standards, and preparing for the needs of an aging population through investments in geriatric respiratory care and health-system capacity. These strategies may help to slow the rising COPD burden in Ghana and support progress toward Sustainable Development Goal 3.4 on reducing premature mortality from non-communicable diseases.

## Data Availability

Publicly available datasets were analyzed in this study. This data can be found here: Institute for Health Metrics and Evaluation's (IHME) GBD 2021 dataset, accessed via the Global Health Data Exchange platform (https://vizhub.healthdata.org/gbd-results/).
