# Supplementary material for: Burden of chronic obstructive pulmonary disease in Ghana and globally from 1990 to 2021, with projections through 2050: a systematic analysis based on the Global Burden of Disease Study 2021
Source: Front Med (Lausanne). 2025 Nov 3;12:1681411. doi: 10.3389/fmed.2025.1681411 (PMC12620206; doi:10.3389/fmed.2025.1681411)
Supplement: Supplementary file 1 [file Table_1.docx]

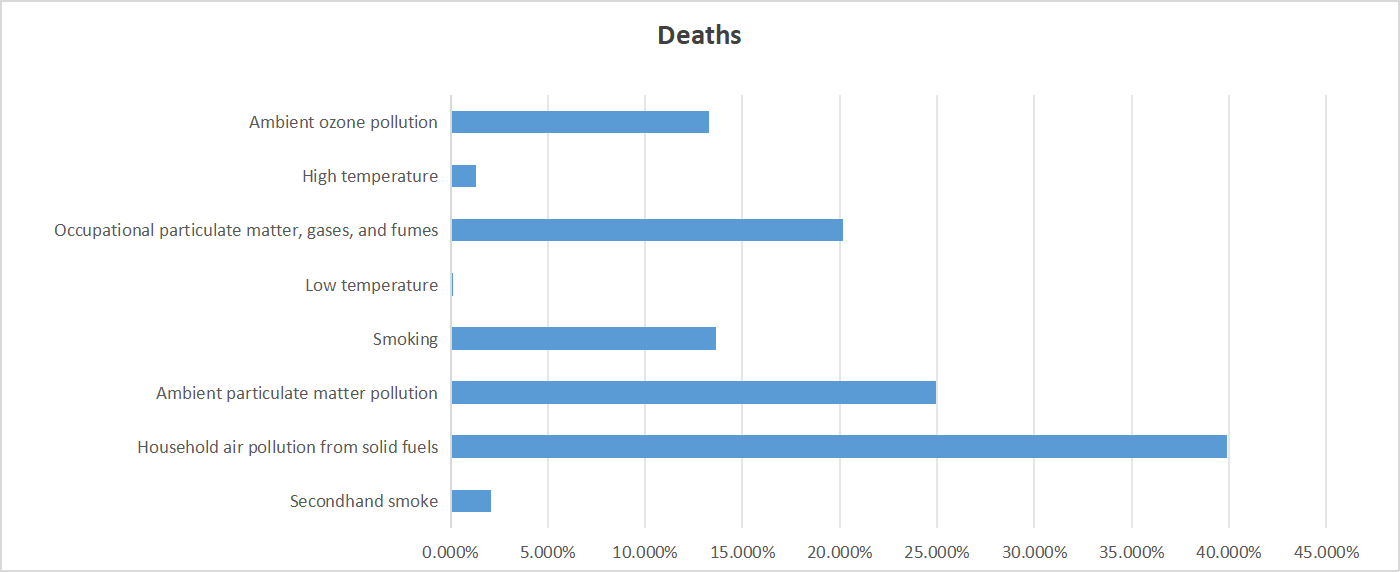


**Figure S1**: Percentage of deaths due to chronic obstructive pulmonary disease (COPD) attributable to all risk factors in Ghana in 2021 (sourced from the Global Burden of Disease Study 2021, <https://vizhub.healthdata.org/gbd-results/).>

The figure presents the percentage of risk factors contributing to COPD deaths in Ghana for 2021. It features a bar chart displaying the proportion of deaths linked to various risk factors, including ambient ozone pollution, high and low temperatures, occupational particulate matter/gases/fumes, smoking, ambient particulate matter pollution, household air pollution from solid fuels, and secondhand smoke.


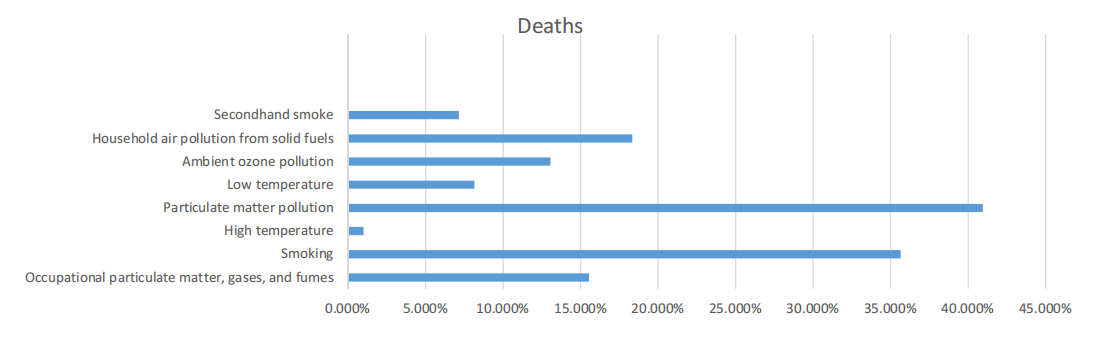


**Figure S2**: Percentage of deaths due to chronic obstructive pulmonary disease (COPD) attributable to all risk factors globally, in 2021 (sourced from the Global Burden of Disease Study 2021, <https://vizhub.healthdata.org/gbd-results/).>

The figure presents the percentage of all risk factors contributing to COPD deaths globally for 2021. It features a bar chart displaying the proportion of deaths linked to various risk factors, including ambient ozone pollution, high and low temperatures, occupational particulate matter/gases/fumes, smoking, ambient particulate matter pollution, household air pollution from solid fuels, and secondhand smoke.


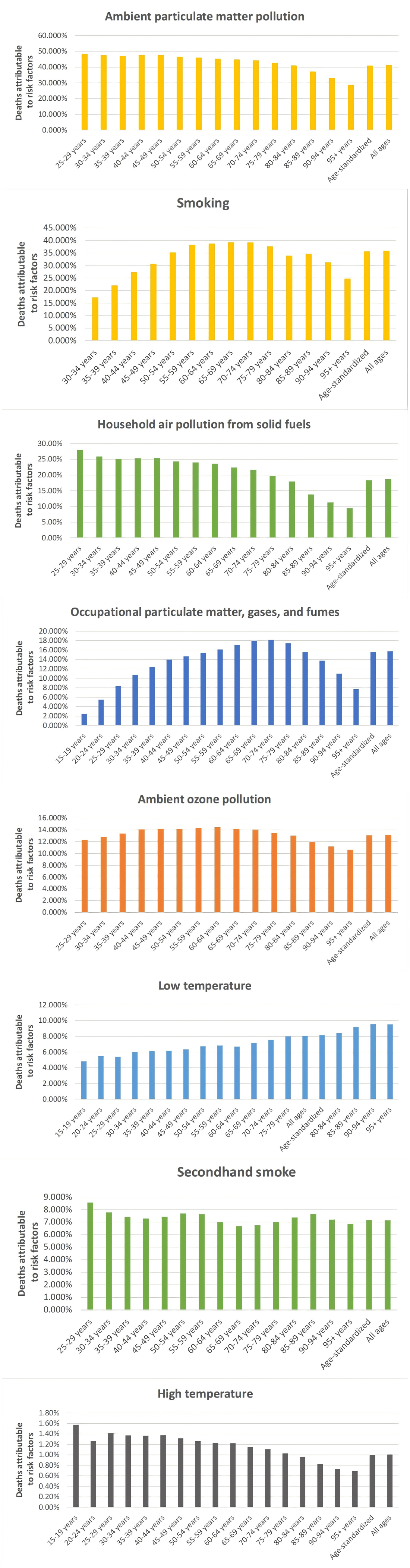


**Figure S3**: Percentage of deaths due to chronic obstructive pulmonary disease (COPD) attributable to individual risk factors globally, in 2021 (sourced from the Global Burden of Disease Study 2021 <https://vizhub.healthdata.org/gbd-results/).>

The figure presents the percentage of risk factors contributing to COPD DALYs globally for 2021. It features a bar chart displaying the proportion of deaths linked to various risk factors, including ambient ozone pollution, high and low temperatures, occupational particulate matter/gases/fumes, smoking, ambient particulate matter pollution, household air pollution from solid fuels, and secondhand smoke.


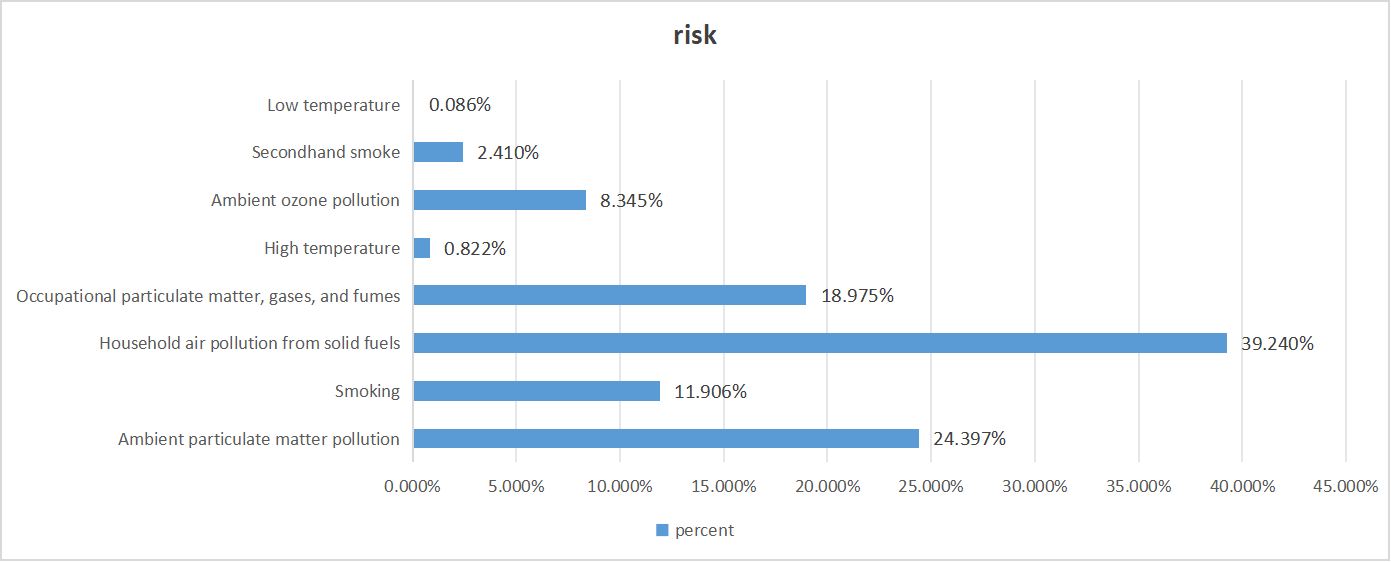


**Figure S4**: Percentage of DALYs from chronic obstructive pulmonary disease (COPD) attributable to all risk factors in Ghana in 2021, sourced from the Global Burden of Disease (GBD) 2021 Study (<https://vizhub.healthdata.org/gbd-results/).>

**
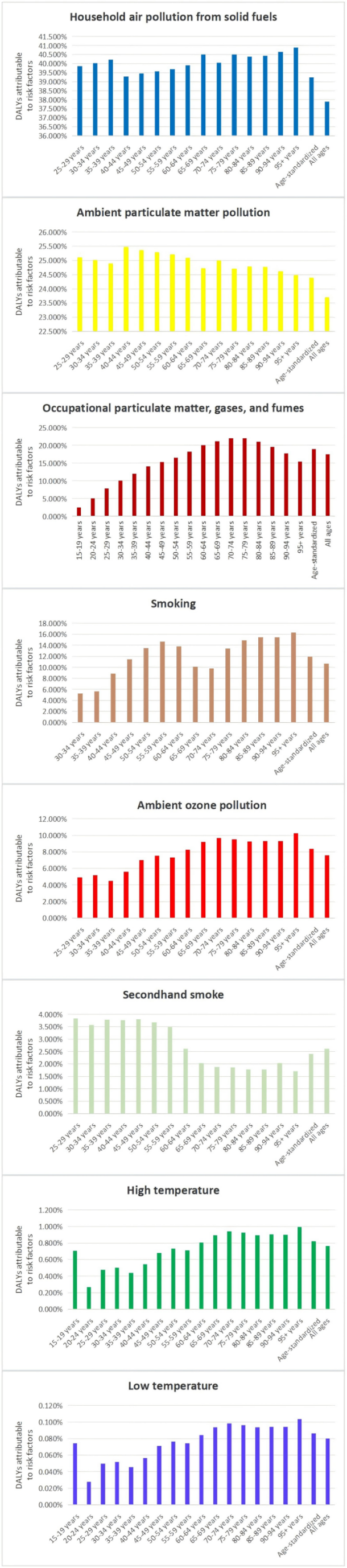
**

**Figure S5:** Percentage of DALYs from chronic obstructive pulmonary disease (COPD) attributable to individual risk factors in Ghana in 2021, soured from from the Global Burden of Disease (GBD) 2021 Study (<https://vizhub.healthdata.org/gbd-results/).>


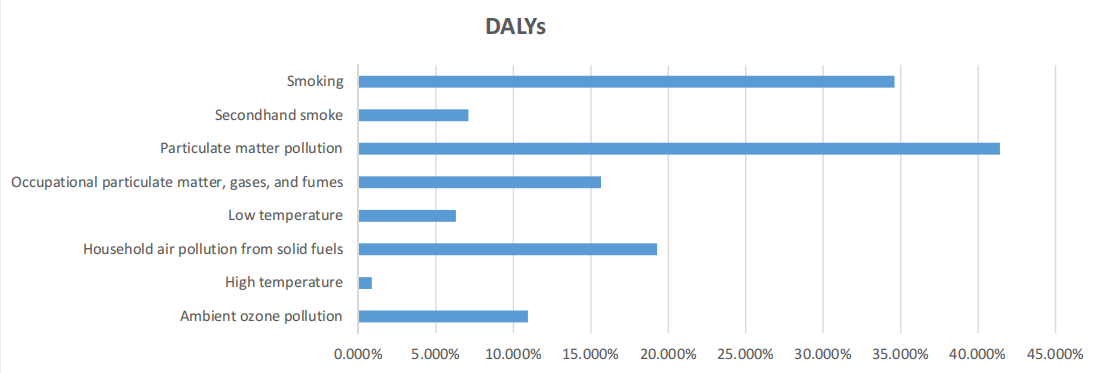


**Figure S6**: Percentage of DALY from chronic obstructive pulmonary disease (COPD) attributable to all risk factors globally in 2021, sourced from the Global Burden of Disease (GBD) 2021 Study (<https://vizhub.healthdata.org/gbd-results/).>

**
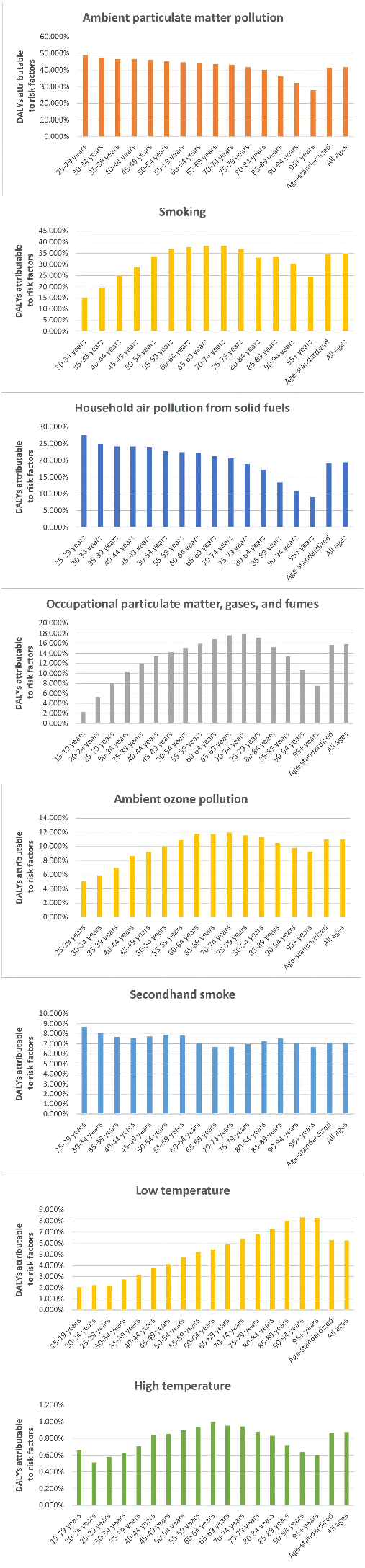
**

**Figure S7**: Percentage of DALY from chronic obstructive pulmonary disease (COPD) attributable to individual risk factors globally in 2021, soured from from the Global Burden of Disease (GBD) 2021 Study ( <https://vizhub.healthdata.org/gbd-results/).>


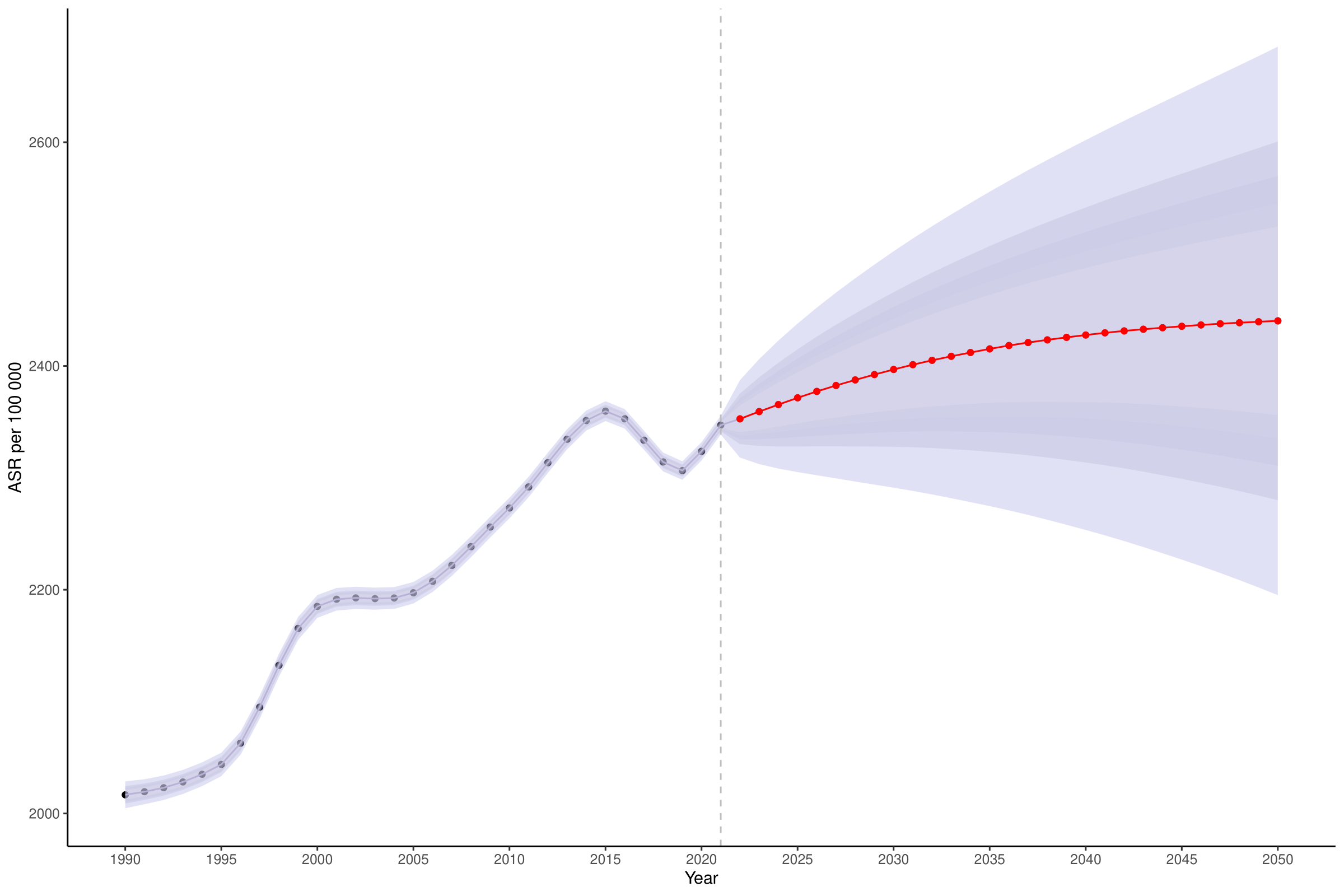


**Figure A8**: Projected COPD ASPR by age in Ghana,1990- 2025: rates per 100,000 Population

This figure presents the projected ASPR for COPD in Ghana from 2025 to 2050, derived from Bayesian forecasting models. The y-axis shows ASPR per 100,000 population. Shaded areas represent uncertainty intervals for the projections.

Abbreviations:

COPD: Chronic Obstructive Pulmonary Disease, a progressive respiratory condition.

ASPR: Age-Standardized Prevalence rate.


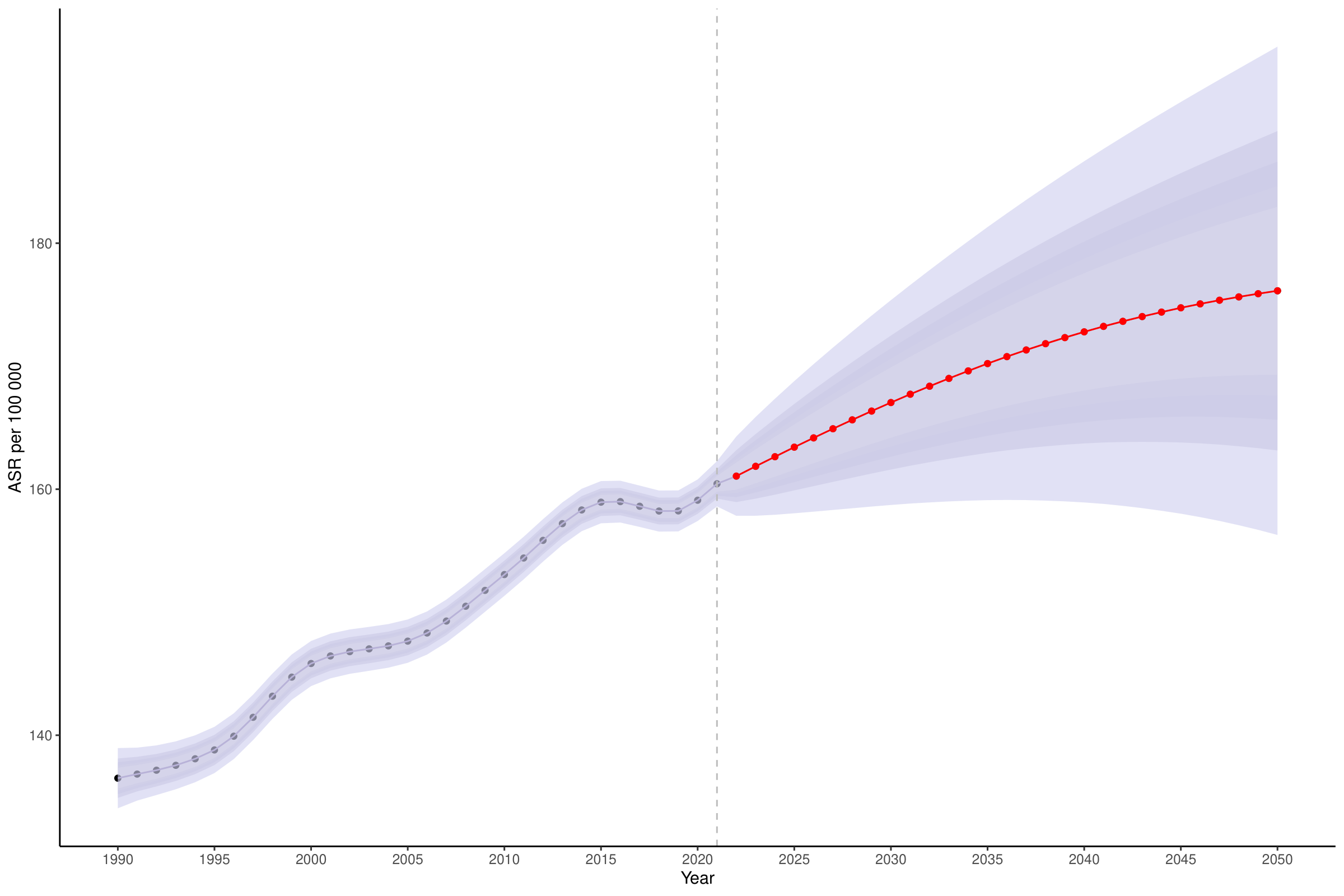


**Figure S9**: Projected COPD ASIR by Age Group in Ghana, 1990–2050

This figure presents the projected ASIR for COPD in Ghana from 2025 to 2050, derived from Bayesian forecasting models. The y-axis shows ASIR per 100,000 population. Shaded areas represent uncertainty intervals for the projections.

Abbreviations:

COPD: Chronic Obstructive Pulmonary Disease.

ASIR: Age-Standardized Incidence rate.


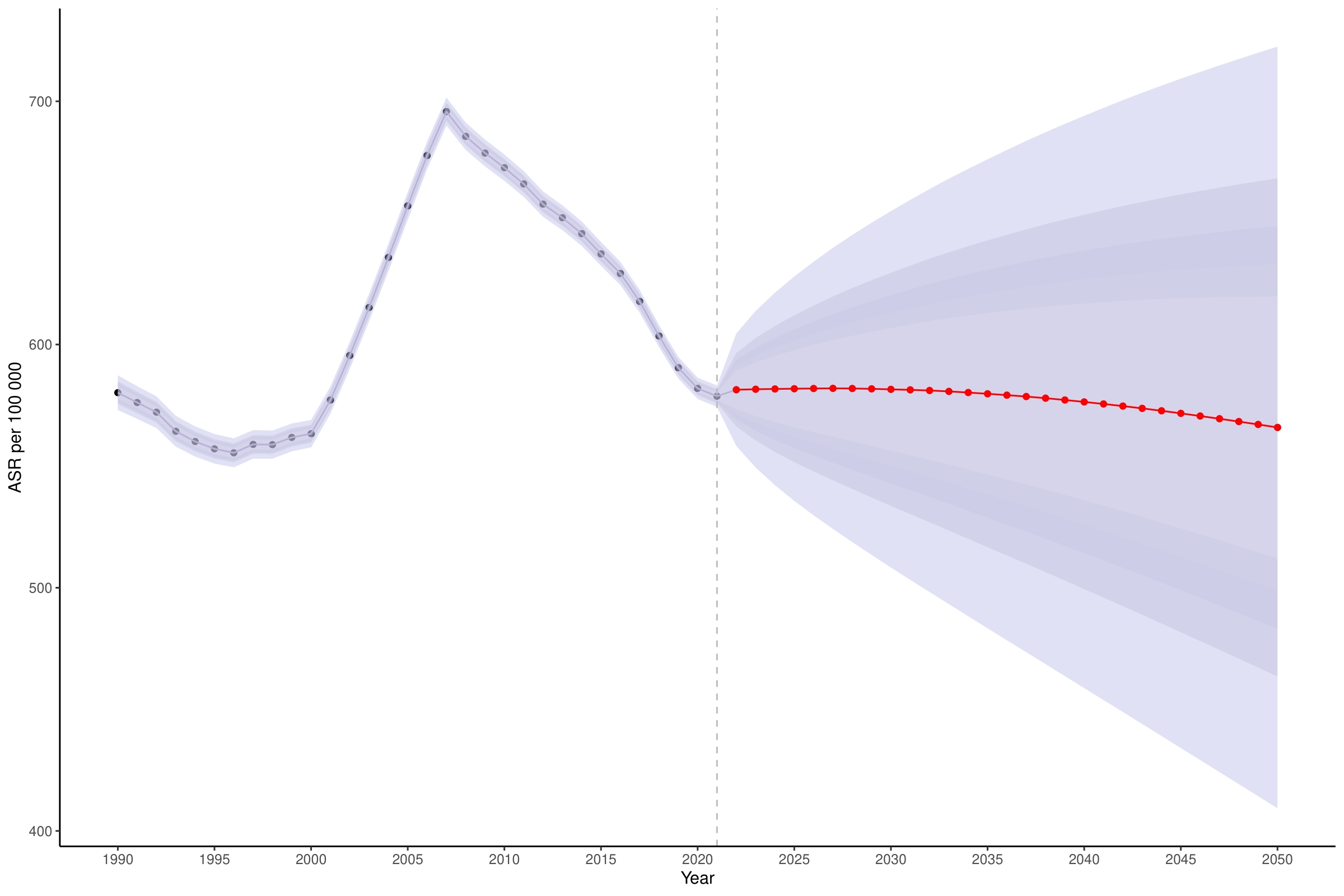


**Figure S10**: Projected Age-Standardized Disability-Adjusted Life Years (ASDALYs) for COPD in Ghana, 2025–2050.
This figure presents the projected ASDALYs for COPD in Ghana from 2025 to 2050, derived from Bayesian forecasting models. The y-axis shows ASDALYs per 100,000 population, with a gradual decline projected over the period. Shaded areas represent uncertainty intervals for the projections.

Abbreviations:

COPD: Chronic Obstructive Pulmonary Disease, a progressive respiratory condition.

ASDALYs: Age-Standardized Disability-Adjusted Life Years.


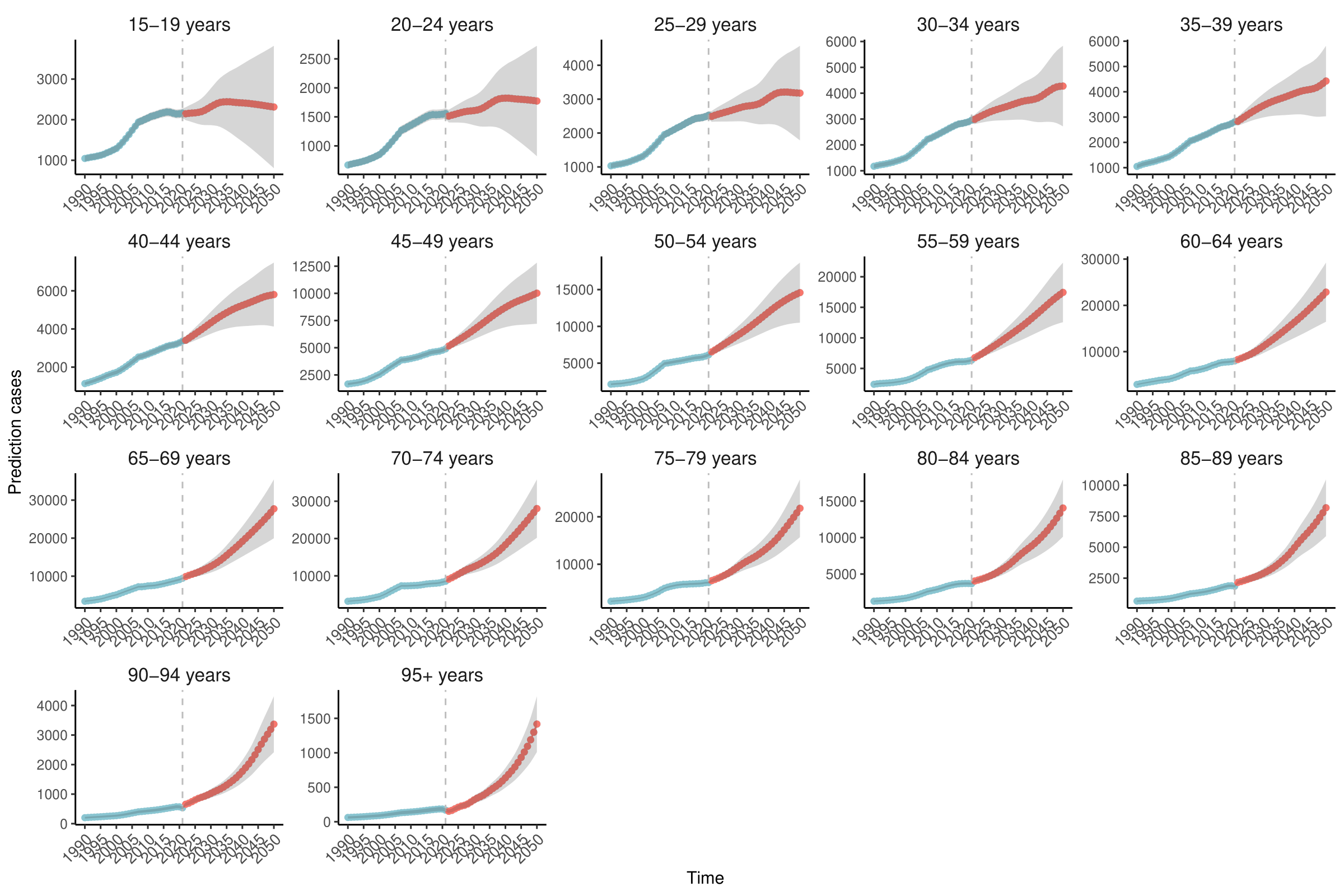


**Figure S11**: Projected Age-Specific Disability-Adjusted Life Years (DALYs) for COPD in Ghana, 2025–2050

This figure presents the projected disability-adjusted life years DALYs by age for COPD in Ghana from 2025 to 2050, stratified by age. The y-axis shows DALYs by number of cases.

Abbreviations:

COPD: Chronic Obstructive Pulmonary Disease, a progressive respiratory condition.

DALYs: Disability-Adjusted Life Years.

Data Source: All figures are derived from the Global Burden of Disease (GBD) 2021 Study (https://vizhub.healthdata.org/gbd-results/).
